# A Soft-Tissue Landmark to Assess Humeral Component Rotation in Total Elbow Arthroplasty

**DOI:** 10.7759/cureus.41729

**Published:** 2023-07-11

**Authors:** Hassan Azimi, Farhan Ahmad, Andre D Sabet, Mark Cohen, Steve Maschke, Robert Wysocki, Xavier Simcock

**Affiliations:** 1 Department of Orthopedic Surgery, Rush University Medical Center, Chicago, USA; 2 Department of Orthopedic Surgery, Cleveland Clinic Foundation, Cleveland, USA

**Keywords:** elbow, total elbow arthroplasty, intermuscular septum, flexion-extension axis, landmark, humeral rotation

## Abstract

Hypothesis: Assessing the rotational alignment of the humeral component during total elbow arthroplasty is dependent upon bony landmarks that can be absent or altered in cases of distal humerus fractures, revision arthroplasty, severe bone loss, or deformity. We hypothesize that the intermuscular septum can be used as a reliable soft-tissue landmark to set the rotation of the humeral component intra-operatively when previously described bony landmarks are not reliable or present.

Materials and methods: Forty-eight unpaired cadaveric human subjects (mean age and standard deviation 63 ± 12 years; 24 males, 24 females) underwent computed tomography (CT) scans. The geometric centers of the trochlea and capitellum were assessed, and the line through these two points was set as the flexion-extension axis (FEA) of the elbow. The intermuscular septum axis (IMSA) was drawn proximal to the olecranon fossa and at least 4 cm proximal to the most distal point of the articular surface, where the posterior humeral cortex was flat. The angles between the FEA and IMSA were calculated and compared using a two-tailed t-test. Regression analysis was used to assess the inter- and intra-observer reliability of the IMSA.

Results: The IMSA was externally rotated 10.3° ± 2.8 compared to the FEA (p < 0.001 and confidence interval (CI) of 2.8 with α set to 0.01). The inter- and intra-observer reliability of the IMSA was high, with an R-value of 0.91 and 0.97, respectively.

Conclusions: The intermuscular septum can be used as a soft-tissue landmark to set humeral component rotation and is 10.3° externally rotated with respect to the FEA of the ulnohumeral joint.

## Introduction

Total elbow arthroplasty (TEA) is an accepted treatment option for patients with end-stage elbow arthritis. Classically, TEA has been used to treat inflammatory arthritis in low-demand patients, in which functional outcomes are most successful [1]. Recent studies have shown that while the frequency of TEA operations has been increasing, the indications for TEA have shifted such that surgery for inflammatory arthritis is decreasing and acute trauma and post-trauma indications are increasing [2, 3]. In addition, incidences of revision TEA have also increased [2]. In cases of trauma, post-traumatic deformity, and revision TEA, the bony landmarks that have been described to orient surgeons in order to implant components in the correct anatomic alignment can be distorted or absent [4]. This can lead to malalignment of implanted components and abnormal kinematics [5]. Malalignment of TEA components places increased loads at the bone-cement interface and can possibly lead to early implant failure [6,7]. A soft-tissue landmark has been described in proximal humerus hemiarthroplasty to correctly orient the surgeon when bony landmarks are not available due to fracture [8]. However, no soft-tissue landmarks have been described to orient surgeons to humeral component positioning during TEA.

The purpose of this study is to test the hypothesis that the intermuscular septum axis (IMSA) is a reliable soft-tissue landmark to set rotation of the humeral component intra-operatively when bony landmarks are not present or are not reliable due to deformity.

## Materials and methods

**Figure 1:**
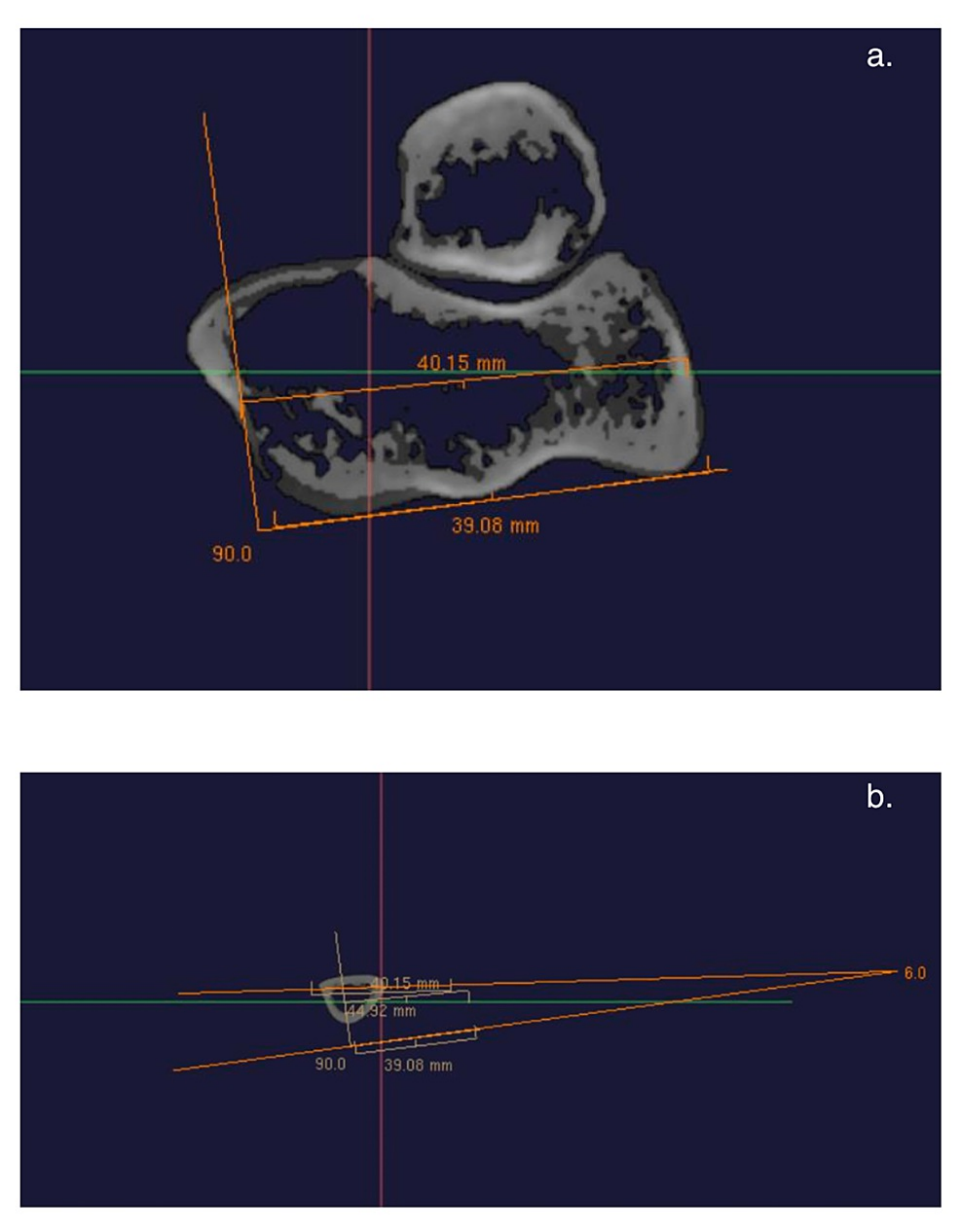
CT measurements of the intermuscular septum. Measurements of the intermuscular septum axis drawn proximal to the olecranon fossa (a) and viewed in the axial plane (b).

Following institutional review board approval (Rush University Medical Center, Chicago, Illinois (ORA#: 20090207-IRB01)), normal cadaveric specimens were obtained and used without any alterations in anatomy. Forty unpaired human computed tomography (CT) scans were obtained (24 males, 24 females; mean age and standard deviation 63 ± 12 years) using a 64-slice clinical scanner (GE LightSpeed Ultra; GE Healthcare, Milwaukee, Wisconsin) with slice thickness set to 0.625 mm. Three-dimensional models were created by importing these images into Arthoplan (Cleveland Clinic Foundation, Cleveland, Ohio). The CT images were oriented to ensure that the slices were perpendicular to the long axis of the humerus. The geometric centers of the trochlea and capitellum were assessed. In accordance with previously described methodologies, the line through these two points was set as the flexion-extension axis (FEA) of the elbow [4,6,7,9]. The intermuscular septum axis (IMSA) was drawn proximal to the olecranon fossa and at least 4 cm proximal to the most distal point of the articular surface, where the posterior humeral cortex was flat (Figure 1a). Using Arthroplan, these lines were converted into two dimensions and superimposed on axial slices to obtain a two-dimensional angle measurement for each CT (Figure 1b).

Descriptive statistics and two-tailed t-tests (p<0.05) were performed on the data collected using Microsoft Excel (Microsoft Corporation, Redmond, Washington). Intra-observer and inter-observer repeatability testing was performed by the fellowship-trained elbow surgeons who radiographically assessed the IMSA. Predictive Analytics Software (PASW) Statistics, version 18.0 (SPSS Inc., Chicago, Illinois), was used to determine reliability. Kappa testing was used to further quantify inter-observer reliability.

## Results

**Figure 2:**
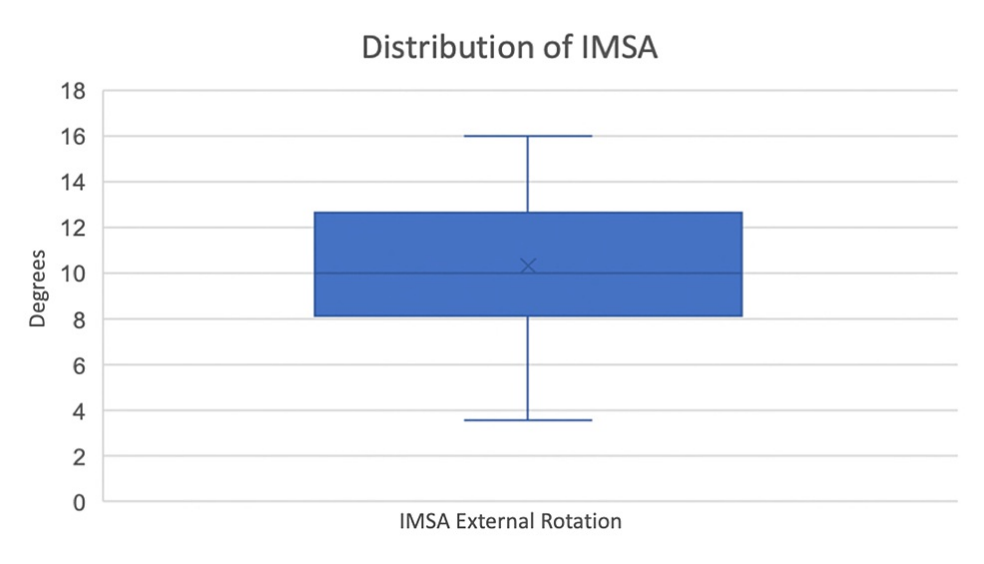
Intermuscular septum axis distribution. Distribution of the measurements of the intermuscular septum axis (IMSA)

The IMSA was externally rotated 10.3° ± 2.8 (mean, standard deviation) compared to the FEA (p < 0.001) and had a confidence interval (CI) of 2.8 with alpha set to 0.01. Figure 2 demonstrates the distribution of measurements for the IMSA.

There were 24 males and 24 females included in the study. The average IMSA for males was 10.1° ± 2.7, and for females, it was 10.6° ± 2.8. There was no statistical difference between these groups (p = 0.75). The inter- and intra-observer reliability of the IMSA was high, with R-values of 0.91 and 0.97, respectively.

## Discussion

**Figure 3:**
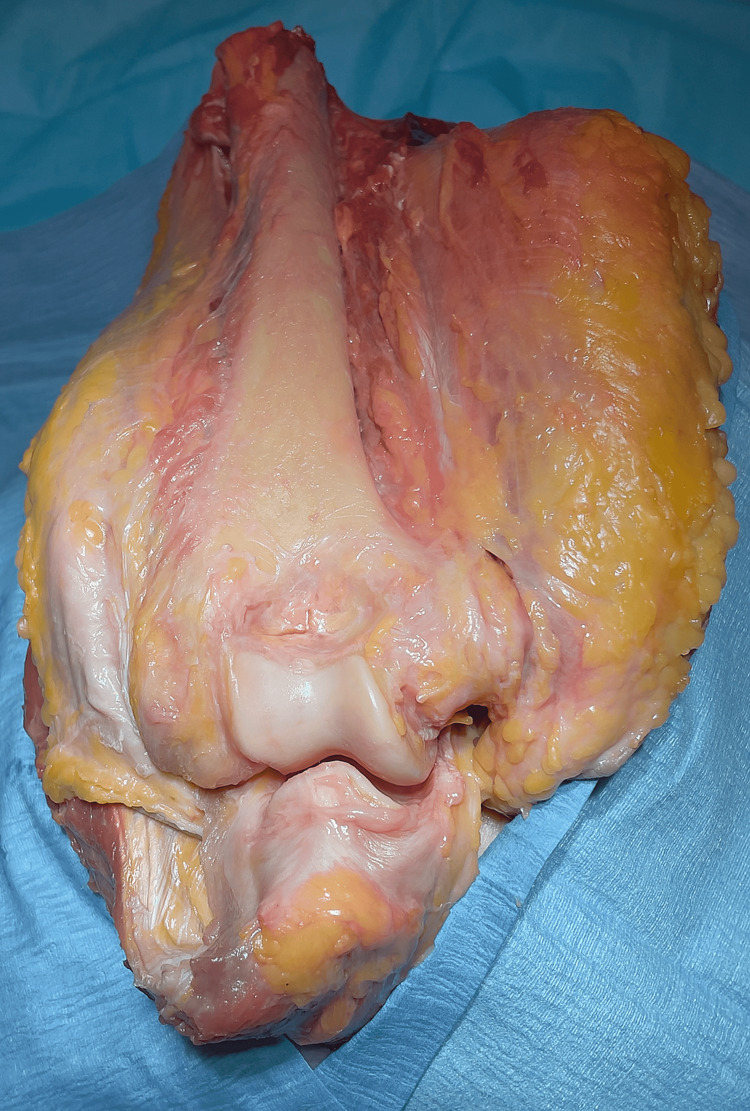
Coronal view of the intermuscular septum axis. A surgeon’s view of the intermuscular septum axis in a cadaveric specimen, tracking both medially and laterally along the humerus.

**Figure 4:**
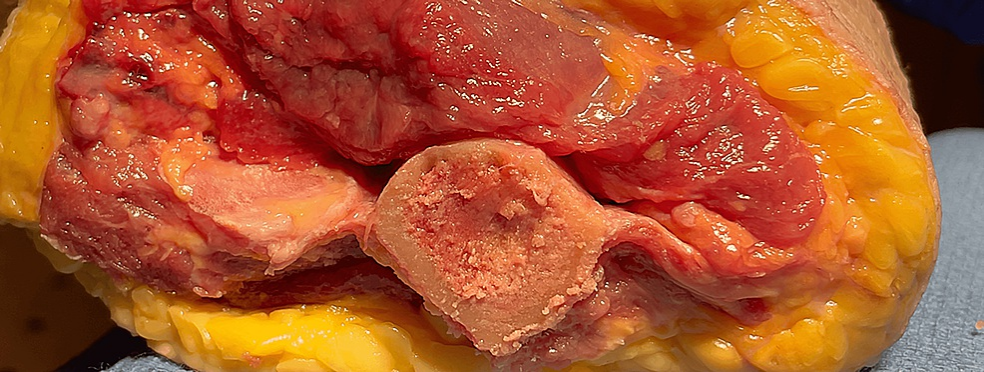
Axial view of the intermuscular septum axis in a cadaveric specimen.

These results support our hypothesis that the intermuscular septum is a soft-tissue landmark that can be used to assess humeral component rotation. While previous authors have described techniques to assess the humeral rotation using bone landmarks, there are no previous descriptions in the literature of soft-tissue landmarks to orient surgeons on the rotational alignment of the humeral component [4,10]. The clinical significance of this is that a soft-tissue landmark can be used to orient surgeons on correct humeral component rotational alignment when bone loss is present (Figures 3-4).

Furthermore, it has been shown that surgeon accuracy in assessing the flexion-extension access (FEA) of the humerus with visual bone landmarks is variable, even with normal anatomy [6]. The intermuscular septum can serve as one additional check to precisely assess the humeral component's rotational alignment.

Our data suggest that the IMSA can be identified with low variability. The IMSA is useful in that it can be added to the arsenal of landmarks used in assessing humeral rotation alignment or can be independently used when previously described bone landmarks are no longer present or reliable due to fracture, bone loss, or deformity. It should be noted that the IMSA does require a correctional factor, similar to the posterior cortical humeral line, and is not parallel to the FEA. However, the IMSA does not differ significantly based on gender, which is an improvement from the posterior humeral cortical line. The inter- and intra-observer reliability of the IMSA was high, with R-values of 0.91 and 0.97, respectively. This suggests that not only is the IMSA a reliable landmark for the individual surgeon who is familiar with it but it can be reliably used by others as well.

Elbow flexion and extension occur through a single axis termed the FEA [11]. The FEA is defined by a line connecting the geometric centers of the trochlear sulcus and the capitellum [6,11]. The ability to intra-operatively determine the FEA is most commonly performed by visual cues from bony surface anatomy. On the lateral surface, the FEA exits at a tubercle where the lateral ulnar collateral ligament originates. On the medial surface, the FEA exits just anteriorly and inferiorly to the medial epicondyle. Brownhill et al. performed a cadaveric study to assess the surgeon’s ability to use surface anatomy to determine the FEA [6]. These authors found that in comparison to a computer-generated FEA, surgeons demonstrated an error of 1.5° ± 3.0 valgus and 1.6° ± 3.3 external rotation. This study was performed on normal cadaveric specimens without any alterations in anatomy and with complete removal of all soft tissues, representing a best-case scenario.

Sabo et al. compared the transepicondylar axis and the posterior humeral cortical line to the FEA in order to determine which landmark is most reliable [4]. The transepicondylar access, another surface anatomy landmark, is commonly used to intra-operatively assess humeral rotation and is defined by a line connecting the apex of the condyles. However, in cases of fracture and bone loss, the condyles may not be present intra-operatively. The posterior humeral cortical line is identified as proximal to the olecranon fossa at the most distal axial image, where the posterior cortex is flat. They found that in comparison to the FEA, the transepicondylar axis was externally rotated 2.8°± 3.5° and the posterior humeral cortical line was externally rotated 14.0°± 4.2°. They also found that males had less external rotation of the posterior humeral cortical line in comparison to females (12.6°± 3.6° and 16.4° ± 5.2°, respectively). Furthermore, the range of external rotation was found to be 6.6° to 23.7°, demonstrating wide variability amongst specimens. The inter-observer reliability kappa value for identifying the posterior humeral cortical line was 0.5 in comparison to 0.3 for the transepicondylar axis. The variability, gender differences, and correction factor needed to use the posterior humeral cortical line as an intra-operative landmark make this less than ideal.

Recent systematic reviews have demonstrated aseptic loosening to be the most common complication for linked, semi-constrained TEA [12,13]. To understand how malalignment contributes to aseptic loosening, ex vivo biomechanical analyses have been performed. Rotational malalignment of the humeral component of 10° places a linked semi-constrained implant at the structural limit of the semi-constrained hinge during flexion extension with a varus or valgus moment [14]. This finding of rotational malalignment raises concerns that implanting the prosthesis in a manner that changes the kinetic pattern to force the hinge to rotate at its structural limit may lead to early failure. Humeral component malalignment has also been shown to place increased loads on the humeral stem in a linked semi-constrained implant [7]. This laboratory study demonstrated that malalignment of the humeral component by just 8° of rotation or 6° of varus or valgus can significantly increase load transfer at the humeral component. Clinical series of implant failures often note polyethylene wear, bushing wear, metallosis, and third-body particulate disease at the time of revision TEA [15,16]. However, these series have not elucidated how malalignment contributes to these wear patterns. A retrospective review of 21 elbows demonstrated that alignment affects functional outcomes and pain scores; however, they did not find a correlation with loosening, likely due to the small number of cases [17]. Given the trend towards an increase in the utilization of TEA as a primary treatment for trauma and post-traumatic disorders, emphasis should be placed on understanding the optimal ways to assess alignment and how it affects implant survival and patient-reported outcome measures.

Strengths of this study include the large number of elbows reviewed to identify a soft-tissue landmark that can be reproducibly identified. Another strength is that the IMSA reproducibly identifies the FEA without a correctional factor, decreasing the complexity of using this landmark intra-operatively. The IMSA is also not significantly different between genders and has high intra- and inter-observer reliability.

One limitation of this study is the use of CT scans to assess the accuracy of this measurement. Further study of cadaveric specimens would better replicate an intra-operative scenario. Another limitation of this study is the use of CT scans of patients with normal distal humerus anatomy that is unaltered by fracture or articular deformity. Despite this, abnormal articular surfaces or distorted anatomy should not affect the ability to make this extra-articular measurement. A further study of patients undergoing total elbow arthroplasty should be performed to validate this measurement.

## Conclusions

Surgeon accuracy in assessing the flexion-extension access (FEA) of the humerus with visual bone landmarks is variable, even with normal anatomy. These bone landmarks are not always present in revision operations, trauma, or with bone loss. The intermuscular septum is a reliable soft-tissue landmark that has low variability among specimens, can be used to assess humeral component rotation, and can serve as a check during surgery to implant the humeral prosthesis more precisely.
